# Prediction of the Outcome for Patients with Glioblastoma with lncRNA Expression Profiles

**DOI:** 10.1155/2019/5076467

**Published:** 2019-12-23

**Authors:** Qinglin Liu, Changjing Qi, Gang Li, Wandong Su

**Affiliations:** ^1^Department of Neuro-intervention, Beijing Neurosurgical Institute and Beijing Tiantan Hospital of Capital Medical University, Beijing 10050, China; ^2^Department of Nursing, Qilu Hospital of Shandong University, Jinan 250012, China; ^3^Department of Neurosurgery, Qilu Hospital of Shandong University, Jinan 250012, China

## Abstract

**Background:**

Progress in gene sequencing has paved the way for precise outcome prediction of the heterogeneous disease of glioblastoma. The aim was to assess the potential of utilizing the lncRNA expression profile for predicting glioblastoma patient survival.

**Materials and Methods:**

Clinical and lncRNA expression data were downloaded from the public database of the cancer genome atlas. Differentially expressed lncRNAs between glioblastoma and normal brain tissue were screened by bioinformatics analysis. The samples were randomly separated into the training and testing sets. Univariate Cox regression, least absolute shrinkage, selection operator regression, and multivariate Cox regression were performed to develop the prediction model with the training set, which was presented as a forest plot. The performance of the model was validated by discrimination and calibration analysis in both the training and testing sets. Patient survival between model-predicted low- and high-risk subgroups was compared in both the training and testing sets.

**Results:**

One thousand two hundred and fifty-five differentially expressed lncRNAs between glioblastoma and normal brain tissues were screened. After univariate Cox regression and the least absolute shrinkage and selection operator regression, a 12 lncRNA constituted prediction model was developed by multivariate Cox regression. Of the 12 lncRNAs, 4 lncRNAs were independent risk factors for patient survival. The areas under the receiver operating characteristic curves of the model for predicting 0.5-, 1-, 1.5-, and 2-year patient survival was 0.788, 0.824, 0.874, and 0.886, respectively in the training set and 0.723, 0.84, 0.816, and 0.773 in the testing set. The calibration curves of the prediction model fitted well. Significant survival disparity was observed between the model dichotomized low- and high-risk subgroups in both the training and testing set.

**Conclusions:**

LncRNA expression signature can predict glioblastoma patient survival, promising lncRNA-based survival prediction.

## 1. Introduction

Glioblastoma (GBM) is the most aggressive diffuse glioma of astrocytic lineage and is considered a grade IV glioma based on the WHO classification [1]. As the most common malignant primary brain tumor, GBM accounts for about 54% of all gliomas and 16% of all primary brain tumors [2]. Despite progress in the current standard treatment strategy, including maximal safe resection, followed by radiotherapy with concomitant and adjuvant Temozolomide (TMZ), the median survival was only 14.6 months [3]. The high intertumoral and intratumoral heterogeneity make the survival of patients with glioblastoma vary greatly [4, 5]. Traditional clinicopathological factors, including histological grade, age, and Karnofsky performance status, have failed to predict the outcome precisely [6]. Advanced sequencing and microarray technologies have paved the way for understanding the interpatient response variability for treatment at the gene expression and regulation level. Gene expression-based molecular signatures have been discovered and used for identifying new glioblastoma subtypes and gained better prognostic significance [6, 7].

It is evident that only less than 2% of the genome encodes proteins and at least 75% is actively transcribed into noncoding RNAs [8]. Although some of the noncoding transcripts are small, most of them surpass 200 nucleotides in length, and they are therefore cataloged as long noncoding RNAs (lncRNAs) [9, 10]. It is estimated that the human genome contains close to 16,000 genes that encode more than 28,000 distinct lncRNA transcripts [9]. LncRNAs are known to play key roles in a broad range of biological processes such as cell differentiation, human diseases, and tumorigenesis [11]. LncRNAs can regulate the expression levels of oncogenes or tumor suppressors through various mechanisms, including chromatin modification, transcriptional control, and post-transcriptional processing and affect various aspects of cellular homeostasis [9]. The expression pattern of lncRNAs is highly tissue and cell type-specific, revealing the potential for accurate molecular cancer subclassification and outcome prediction [9]. Although an increasing number of lncRNAs have been characterized, the functions of most lncRNAs are still unrevealed [12].

Reports have revealed that lncRNAs are aberrantly expressed in glioma tissue to normal tissue and even in GBM to low-grade gliomas [13]. A variety of lncRNAs have been reported to participate in glioblastoma proliferation, invasion, migration, radioresistance, and immune exemption, providing new targets for gene therapy [14, 15]. Furthermore, lncRNAs have also been proposed as predictors for patient survival [6, 16].

In this study, lncRNAs expression profiles in GBM and normal tissue and clinical data were downloaded from the public database of the cancer genome tlas (TCGA). Different lncRNA expression profiles were compared with bioinformatics analysis. Univariate Cox regression, least absolute shrinkage and selection operator (LASSO), and multivariate Cox regression were conducted to screen the lncRNAs associated with patient survival. A forest plot was developed based on the results from multivariate Cox regression, and the performance of the model was validated.

## 2. Materials and Methods

### 2.1. Dataset Preparation

The lncRNAs expression profile data were downloaded from TCGA (https://cancergenome.nih.gov/) using the TCGAbiolinks package in R (version 3.6. 0, http://www.r-project.org) [17]. lncRNAs expression levels were summarized into transcripts per kilobyte million (TPM) values. The data consisted of 172 samples, of which 5 were normal tissue, and the rest 167 were GBM. The clinical data were also downloaded from the TCGA database and matched to the lncRNA expression signatures. The 167 GBM samples were randomly separated into the training set (120 cases) and the testing set (47 cases). The training set was used for prediction model development and the testing set was for model validation.

### 2.2. Screening the Differentially Expressed lncRNAs

To find the differentially expressed lncRNAs between GBM and normal tissue, differentially expressed genes (DEGs) analysis was conducted using the edgeR package in R. Only lncRNAs with *P* < 0.005 and log value of fold of change larger than 2 were defined as significantly expressed lncRNAs and considered as candidate lncRNAs for further bioinformatics analysis.

### 2.3. Development of the Prediction Model

The development of the prediction model was based on the training set. The LASSO Cox regression model was used to select the most prognostic lncRNAs for overall survival (OS). In brief, univariate Cox regression was conducted to screen the candidate lncRNAs, and only variates with a *P* < 0.05 were enrolled for LASSO regression. Second, LASSO regression was performed to further diminish the variates for multivariate Cox regression. Finally, the variates from LASSO regression were taken into multivariate regression and the prediction model was constructed. The prediction model was demonstrated as a forest plot. The odds ratio (OR) of each variate and the *P* value were demonstrated.

### 2.4. Validation of the Prediction Model

Validation of the prediction model was conducted by discrimination and calibration assays in both the training and testing sets. The predictive accuracy of the lncRNA forest plot was examined by time-dependent receiver-operating characteristic (ROC) analysis. The area under the curves (AUCs) at the different cutoff time was used to measure the predictive accuracy. Calibration curves were produced by plotting the observed rates against the forest plot-predicted probabilities. All the patients were subgrouped into the low- and high-risk subgroups according to the model predicted risks (the cutoff value for discriminating high- and low-risk subgroups was the median model predicted risk of the training set), and survival curves were built and compared between these groups. Patients were also dichotomized into subgroups according to the expression levels of lncRNAs that were independently associated with patient survival, and survival curves were compared between these groups.

### 2.5. Statistical Analysis

All statistical analyses were performed using R (version 3.6.0, http://www.r-project.org). For univariate and multivariate analysis, Cox regression was used. The concordance index (C index) was calculated to evaluate the discrimination capacity of the lncRNAs constructed forest plot. Calibration plots were generated to assess the consistency between the actual outcomes and the forest plot predicted outcomes. The *x*-axis represents the prediction calculated by the forest plot, and the *y*-axis represents the actual outcomes. The Kaplan–Meier method was used to build the survival curves, and the log-rank test was used to test the difference of the survival curves between groups. All statistical tests were 2-sided, and *P* value <0.05 was considered statistically significant. The main packages used included caret, rms, survival, foreign, timeROC, glmnet, survminer, pheatmap, ggplot2, and edgeR.

## 3. Results

### 3.1. Differentially Expressed lncRNAs between GBM and Normal Brain Tissue

**Figure 1:**
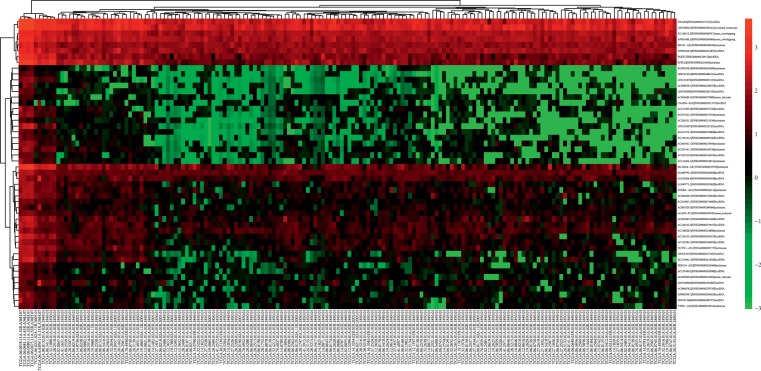
Heatmap showed the top 50 differentially expressed lncRNAs between GBM and normal brain tissue.

**Figure 2:**
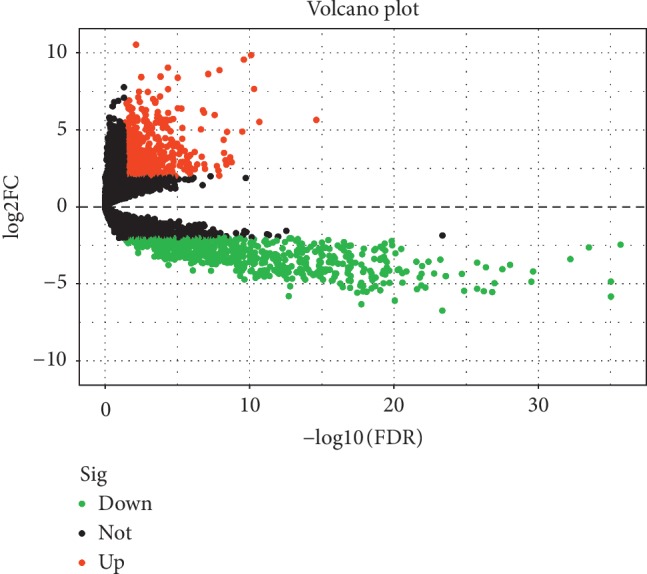
Volcano plot showed the differentially expressed lncRNAs between GBM and normal brain tissues. Black dots: not differentially expressed lncRNAs, Green dots: downregulated lncRNAs with expression level *P* < 0.05 and fold of change >2 and red dots: upregulated lncRNAs with expression level *P* < 0.05 and fold of change >2.

DGEs analysis revealed that, of the 8225 genes enrolled for differentially expression analysis, 1255 lncRNAs were differentially expressed between GBM and the normal brain tissues. Of the 1255 differentially expressed lncRNAs, 591 were upregulated and the rest 664 were downregulated. The top 50 differentially expressed lncRNAs were demonstrated in Figure 1. Distribution of all the differentially expressed lncRNAs was shown in Figure 2.

### 3.2. Development of the Prediction Model

**Figure 3:**
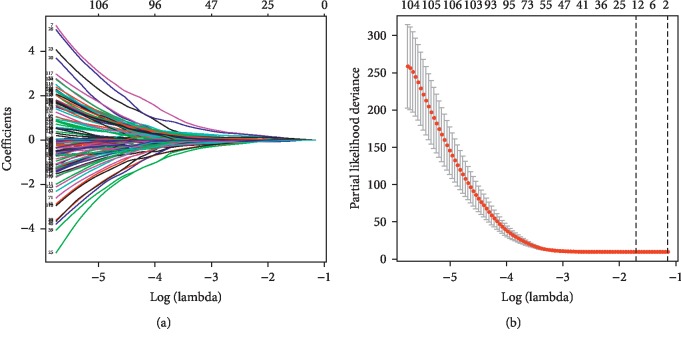
Feature selection using the least absolute shrinkage and selection operator (LASSO) binary logistic regression model. LASSO coefficient profiles of the 132 differentially expressed lncRNAs (a). The tuning parameter (*λ*) selection in the LASSO model used 10-fold cross-validation via minimum criteria (b). A coefficient profile plot was produced against the log (*λ*) sequence. The vertical lines were drawn at the value of the minimum and minimum + 1 standard error selected using 10-fold cross-validation, where optimal *λ* resulted in 12 nonzero coefficients.

**Figure 4:**
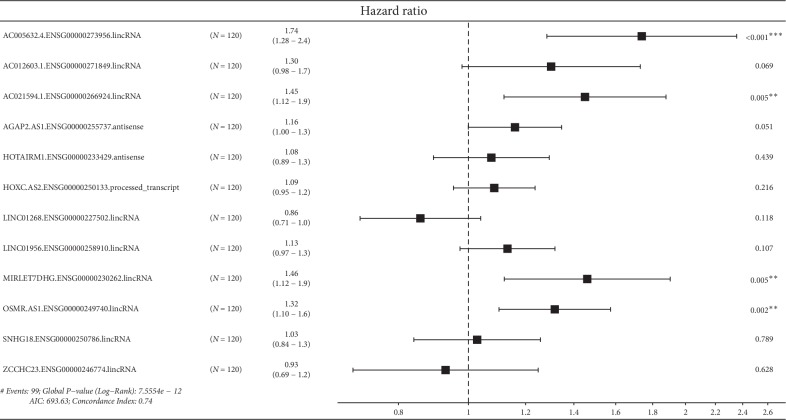
The constitution of the prediction model demonstrated as a forest plot. Four lncRNAs were independent risk factors for patient survival.

Univariate Cox regression revealed that 132 lncRNAs were significantly associated with patient survival. These lncRNAs were enrolled in LASSO Cox regression, and 12 lncRNAs were selected into the multivariate Cox regression. LASSO coefficient profiles of the 132 lncRNAs are presented in Figure 3(a). The tuning parameter (*λ*) selection in the LASSO model used 10-fold cross-validation via minimum criteria (Figure 3(b)). The results of multivariate Cox regression was demonstrated in Figure 4. Of the 12 lncRNAs enrolled in the prediction model, 4 lncRNAs were independent risk factors for patient survival, including AC005632.4.ENSG00000273956.lincRNA, AC021594.1.ENSG00000266924.lincRNA, MIRLET7DHG.ENSG00000230262.lincRNA, and OSMR.AS1.ENSG00000249740.lincRNA.

### 3.3. Validation of the Prediction Model

**Figure 5:**
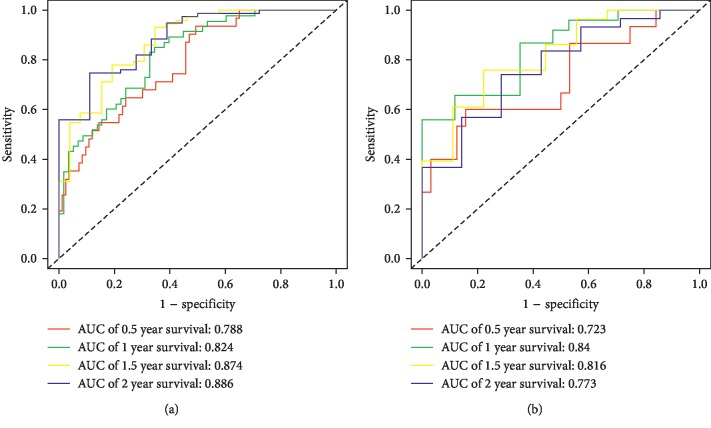
Performance of the prediction model. Receiver-operating characteristic curves (ROCs) for the model in predicting the 0.5-, 1-, 1.5-, and 2-year survival were built, and the prediction accuracy was demonstrated with areas under the curves (AUCs) in the training (a) and testing set (b).

**Figure 6:**
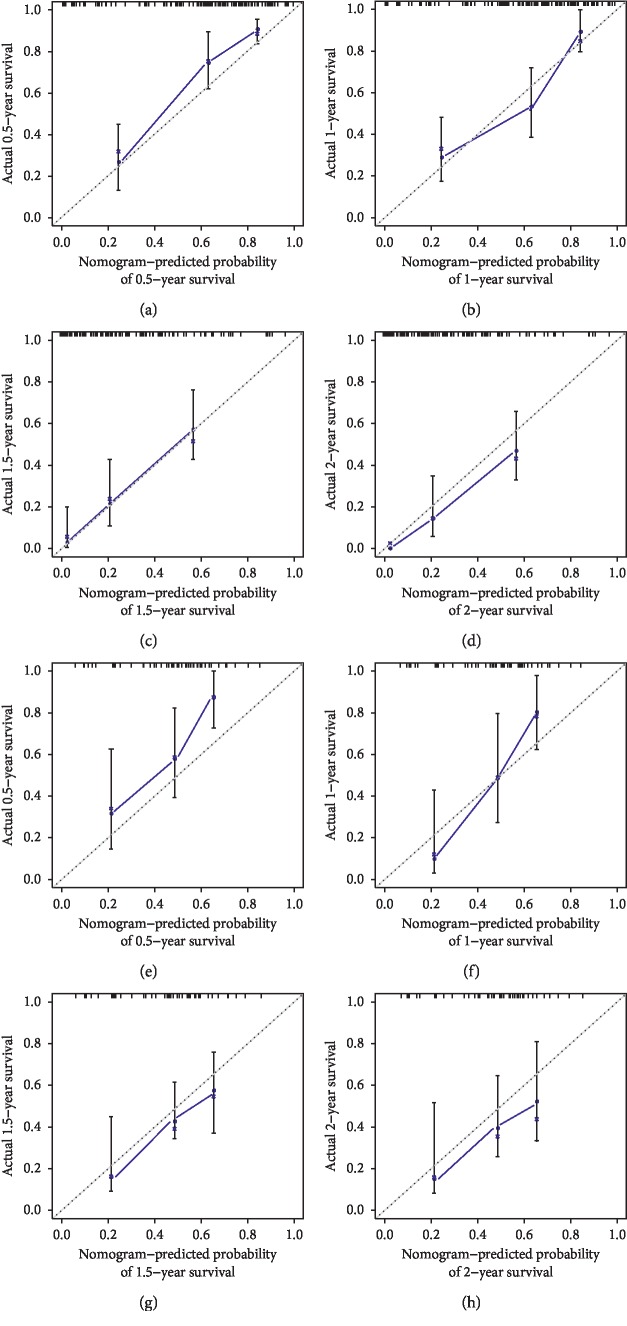
Calibration plots of the model for predicting 0.5- (a), 1- (b), 1.5- (c), and 2-year (d) survival in the training set. Calibration plots of the model for predicting 0.5- (e), 1- (f), 1.5- (g), and 2-year (h) survival in the testing set.

**Figure 7:**
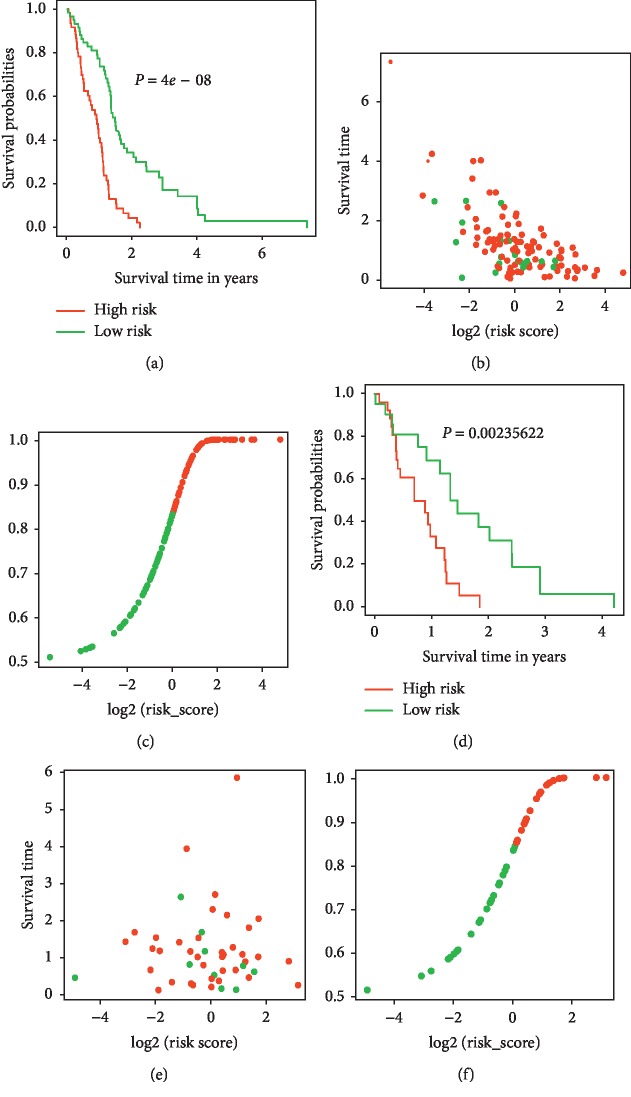
Risk score analysis of the patients. Survival analysis of the GBM patients dichotomized by the model-predicted risk scores in the training (a) long-rank test (*P* < 0.01) and testing set ((d) long-rank test, *P*=0.002). The patient's status along with model-predicted risk scores in the training (b) and testing set (e). Model-predicted risk score distribution of GBM patients in the training (c) and testing (f) set.

Validation of the prediction model was conducted with discrimination and calibration assay. The risk probability of each patient was calculated according to the prediction model, and the ROCs were built for assessing the accuracy of the prediction model. As Figure 5(a) showed, the AUC of the model for predicting the 0.5-, 1-, 1.5-, and 2-year survival was 0.788, 0.824, 0.874, and 0.886, respectively, in the training set and 0.723, 0.840, 0.816, and 0.773 in the testing set, demonstrating a reasonable prediction accuracy. The C-index of the prediction model was 0.743 (95% CI 0.716–0.77) in the training set and 0.707 (95% CI 0.670–0.745) in the testing set. The calibration curves for the prediction of survival demonstrated acceptable agreement between the prediction and observation in the primary cohort (Figure 6). All the patients were subgrouped into the high and low-risk groups, and the survival curves were built and compared (Figures 7(a) and 7(d)). The high-risk group showed a significant poor survival status than the low-risk group in both the training (Figure 7(a), *P* < 0.001) and testing group (Figure 7(c), *P*=0.002), implying the significance of our model in clinical practice for discriminating the risks for survival. A comparison of risk score with patient survival status and risk score distribution among GBM patients are shown in Figures 7(b) and 7(c) for the training set, and Figures 7(e)–7(f) for the testing set.

### 3.4. Survival Analysis between High and Low Expression lncRNAs Subgroups That Related to Overall Survival

**Figure 8:**
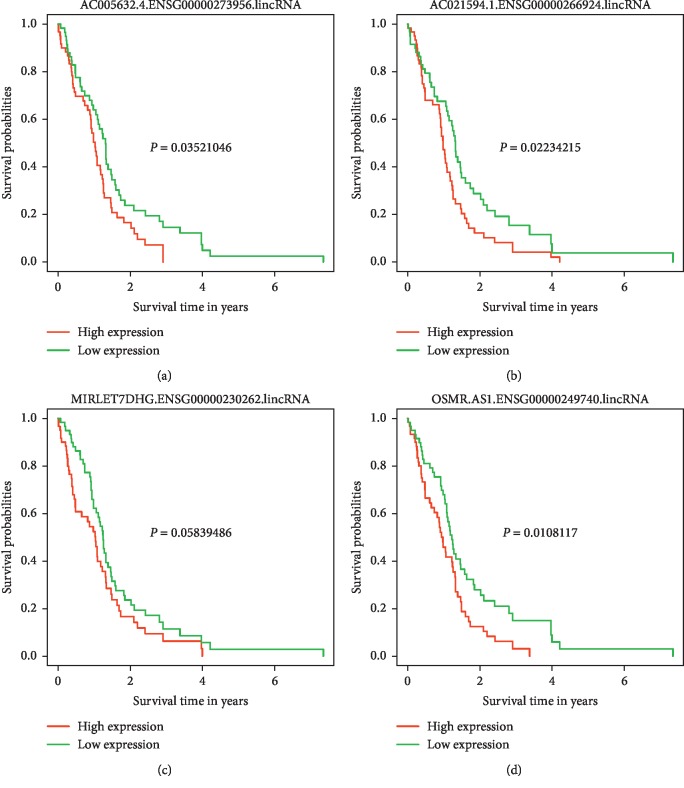
Survival analysis of GBM patients dichotomized by independent lncRNAs associated with overall survival in the training set. Survival analysis of the GBM patients dichotomized by AC005632.4.ENSG00000273956.lincRNA (a). Survival analysis of the GBM patients dichotomized by AC021594.1.ENSG00000266924.lincRNA (b). Survival analysis of the GBM patients dichotomized by MIRLET7DHG.ENSG00000230262.lincRNA (c). Survival analysis of the GBM patients dichotomized by OSMR.AS1.ENSG00000249740.lincRNA (d).

Patients were categorized into low and high expression subgroups according to the expression level of lncRNA that were independent risk factors for overall survival. Survival curves of the high and low expression subgroups for each lncRNA were constructed and compared. As shown in Figure 8, survival was significantly different between the high and low expression subgroups categorized by AC005632.4.ENSG00000273956.lincRNA, AC021594.1.ENSG00000266924.lincRNA, and OSMR.AS1.ENSG00000249740.lincRNA.

## 4. Discussion

The high heterogeneity of GBM results in the great variance of patient outcome, thus making an accurate prediction of the prognosis of a certain patient with clinical and pathological findings seem unreliable. New advances in gene sequencing make precise medicine and prediction promising. In this study, we compared the lncRNA expression profiles between GBM and normal brain tissues and found a 12 lncRNA expression profile could predict the patient outcome effectively. These results approved the potential of lncRNA expression in precision diagnosis and prediction.

It has been increasingly recognized that lncRNAs are important components in regulating gene expression, thus affecting vital physiological and pathological processes such as tumor biology. In this study, we firstly compared lncRNA expression profiles in GBM and normal brain tissues with the public database of TCGA. A total of 1255 differentially expressed lncRNAs were selected from 8225 genes detected (Figures 1 and 2). The differentially expressed lncRNAs were selected for outcome prediction for patients with GBM.

Traditionally, the determination of prognosis of this disease was mainly based on the histological classification combined with patient age and tumor size and location [18]. These factors have all been defined as indicators of patient survival and treatment outcome, but these factors have failed to predict the outcome precisely [6]. Survival variability has been observed in cases of glioblastoma with similar clinical and histological features [6]. Characterization of specific genetic alterations using advanced sequencing and microarray technologies have resulted in gene expression-based molecular signatures, and these signatures have unraveled better prognostic significance [6, 7]. Epidermal growth factor receptor amplification, IDH1 mutation, and 1p/19q loss of heterozygosity could segregate distinct molecular subgroups and predict the outcome more precisely than pathological types [6]. Hypermethylation of the MGMT promoter leads to lower expression levels of MGMT, which sensitizes GBM tumors to chemotherapeutic treatment and thus predict a significantly better patient outcome [19]. The high miR-10b level has been observed to confer a poor survival for GBM patients [20]. A ten miRNA expression signature has been proposed for predicting the overall survival in GBM patients [21]. In this study, we proposed a 12 lncRNA expression signatures to predict the overall survival for GBM patients, and the prediction model demonstrated reasonable accuracy and calibration properties. Distinct survivals were observed between our model discriminated high- and low-risk subgroups. Our results further indicated the role of lncRNAs in glioblastoma progression, thus opening the door for the functional study of lncRNAs in GBM.

A total of 12 lncRNAs expression was enrolled in our final prediction model. Of the 12 lncRNAs, 2 were protective lncRNAs, and the rest 10 were risk lncRNAs for patient survival. Four lncRNAs were independent factors with patient survival, and all of them were risk factors. The 4 independent risk factors for patient survival were AC005632.4.ENSG00000273956.lincRNA, AC021594.1.ENSG00000266924.lincRNA, MIRLET7DHG.ENSG00000230262.lincRNA, and OSMR.AS1.ENSG00000249740.lincRNA. As lncRNAs evolve more quickly than protein-coding RNAs, functional prediction by genomic comparison is difficult. Besides, a lack of collateral information also hampers lncRNA function prediction. In spite of many efforts, the lncRNAs with known function remains scarce, and efficient prediction of lncRNA functions is still a considerable challenge [22].

AC005632.4.ENSG00000273956.lincRNA, AC021594.1.ENSG00000266924.lincRNA, and MIRLET7DHG.ENSG00000230262.lincRNA were novel lncRNAs without functional analysis. OSMR.AS1.ENSG00000249740.lincRNA encodes antisense RNA of oncostatin M receptor (OSMR), inhibiting its transcription and OSM signaling. Oncostatin M (OSM) is a multifunctional cytokine that serves several physiological and pathological functions. At the molecular level, OSM can directly or indirectly participate in tumorigenesis and insulin resistance development [23]. Although OSM was initially found to be antiproliferative in tumors, numerous tumorigenic roles for OSM have been reported in a variety of cancers [23]. In cervical carcinoma, OSM exerts several promalignant effects, including a proangiogenic phenotype and increased cell migration and invasiveness [24]. Furthermore, OSM induces M2 polarization of macrophages in the hypoxic tumor microenvironment and promotes tumor growth and metastasis in breast tumors [25]. In glioblastoma, OSM can cease tumor proliferation and instigate astrocytal differentiation by arresting cell cycle in G1 phase [26]. In another study, Natesh et al. found OSM-mediated signaling contributes to aggressive nature associated with mesenchymal features via STAT3 signaling in glioma cells [27]. Consistent with this result, bioinformatics analysis revealed that high OSMR expression indicated poor survival for glioblastoma patients [28]. In a more recent study, OSMR was found to contribute to local immune response and extracellular matrix progress in GBM and was an independent predictive factor for progressive malignancy and prognostic marker in the response prediction to radiotherapy and chemotherapy [29]. In this study, we found the antisense RNA of OSM was an independent risk factor for patient survival. All these results indicate the key role of OSM signaling in glioma progression and invasion, despite some contradictions of these studies. A full illustration of the regulation mechanism of this pathway in glioblastoma is needed in the future. Further functional studies with these novel lncRNAs screened in our study are empirical to fully elucidate the underlying mechanisms of how these lncRNAs determine patient survival.

The 12 lncRNA signature identified in this study classifies patients successfully into low-risk and high-risk groups. This may help clinicians to identify patients belonging to the high-risk group for more effective adjuvant therapy in addition to the standard treatment protocol. We also found that the 4 lncRNAs were independent predictors of GBM patient survival. Further studies are needed to elucidate the function of these lncRNAs.

## 5. Limitations

This study has some limitations. First, only the expression profile of lncRNAs was enrolled in analysis, and other defined risk factors for survival were omitted, such as operation, chemotherapy, and pathological grade. Second, only data from TGCA were enrolled in this study, other cohorts were needed for external validation before the model could be used in clinical practice. Third, only 5 normal brain tissues were analyzed in this study, which may induce bias in determining the DEGs.

## 6. Conclusions

In conclusion, we have developed a 12 lncRNA signature models that can predict GBM patient survival. This result will inspire the research of lncRNAs for understanding the mechanism of GBM genesis and progression. Expression and function analysis of lncRNAs will aid precise diagnosis and outcome prediction for this highly heterogeneous disease.

## Data Availability

The data used in this manuscript were downloaded from the public database of TCGA.
